# Body Image and its Role in Physical Activity: A Systematic Review

**DOI:** 10.7759/cureus.13379

**Published:** 2021-02-16

**Authors:** Cassidy M Foley Davelaar

**Affiliations:** 1 Orthopedics, Nemours Children's Health System, Orlando, USA

**Keywords:** body image, physical activity, physical literacy, sports attrition, youth sports

## Abstract

Despite the benefits of youth sports, most children drop out by the age of 13 years. A better understanding of the etiology of sports dropout in children will serve to improve interventions to keep children active. The objective of this review was to investigate the associations between body image and perceived physical competence and sports attrition in children. A systematic literature review was conducted using PubMed and MEDLINE database searches in compliance with Preferred Reporting Items for Systematic Reviews and Meta-Analyses (PRISMA). Included studies were full-text English articles that addressed body image or perceived competence and attrition or dropout with subjects 21 years and younger. The results were organized by age to demonstrate the changing relationship body image has on physical activity. Evidence revealed that children younger than 7 years have an inflated self-perception and are eager to participate in activities regardless of competence. Between 7 to 10 years of age, children begin to more accurately perceive their skills and draw comparisons with their peers. Elevated body mass index (BMI) becomes a deterrent between 6 to 11 years. After 12 years, teasing and gender identification issues become causes of attrition. In adolescence, body image becomes a significant determinant of continuation of physical activity, more than actual skill. Perceived physical competency and body image do not appear to affect sports attrition in children younger than 7 years. As children get older, BMI/body image and physical competency become greater factors in sports attrition, with body image playing a significant role in adolescents.

## Introduction and background

The benefits of youth sports participation are numerous and uncontested. Motor competence in children is positively associated with cardiorespiratory fitness, muscular strength, muscular endurance, and a healthy weight status [1]. Research has proven that children who play sports are more likely to incorporate health and fitness into their adolescent lives [2,3]. In addition to their physical benefits, sports should be fun, and the exercises involved in participation should assist in the development of movement skills. Fundamental movement skills are movements that are the building blocks for more complex physical activities like sports. Examples of these skills include object control skills (throwing, kicking, striking, catching, underhand rolling, and dribbling) and locomotor skills (running, hopping, jumping, sliding, leaping, and galloping). The acquisition and gradual mastery of these skills lead to the development of physical literacy. Somewhat similar to academic literacy, physical literacy is important to the motor development and the physical, cognitive, and social growth of children [4].

Despite the benefits of developing motor competence at a young age, 70% of children will drop out of sports by age 13 years. By age 14 years, girls drop out of sports at a rate two times greater than that of boys [5]. “Not fun” followed by “not good enough” are often cited as top reasons for sports attrition [6-8]. However, diving deeper into the literature, “fun” and “not good enough” are very subjective and are packed with deeper meaning. Children will drop out of sports because of their perception of competence, even when competency is not measured [8]. The deeper meaning to sports attrition, children’s perceptions, and self-assessment fueled this research into the role body image plays in sports attrition. Negative body image and poor self-esteem affect physical activity in a similar way as poor perception of skills [7-10]. By looking at the data in a new way, organized by children’s ages, new information regarding causes and timing of sports attrition will be gained as well as new knowledge as to how to improve children’s and adolescents’ physical activity levels. With that, we set out to answer the following questions: 1) what are the specific factors that affect sports attrition in children and adolescents, and 2) does body image and self-perception of physical competency vary by age with regard to sports attrition in children and adolescents?

## Review

A systematic review of the literature following Preferred Reporting Items for Systematic Reviews and Meta-Analyses (PRISMA) guidelines was conducted by a medical librarian. A PubMed (MEDLINE) search was initially performed to collect applicable literature using the terms “body image” AND “young athletes.” This process was repeated every six months from February 2017 to April 2020 to include new references. The reference section for each full-text article was reviewed for additional inclusions. Articles were included if their demographics were of a pediatric population (average 21 years and younger) and if they were in English. Synonyms “drop out” and “perceived competence” were included to obtain best-fit articles. “Perceived competence” is the term used in the youngest ages of our results. We interpreted “perceived competence” to be similar to the definition of body image: an individual’s psychological experience of the function of his or her own body [11]. Articles were excluded if there was only an abstract available, the paper was a review, or the paper did not address physical activity and sports attrition or drop out. There were many articles supporting overweight children with excessive weight being a factor in their continuation or involvement in physical activity. In this case, only articles that reported novel results were included to avoid redundancy.

**Figure 1:**
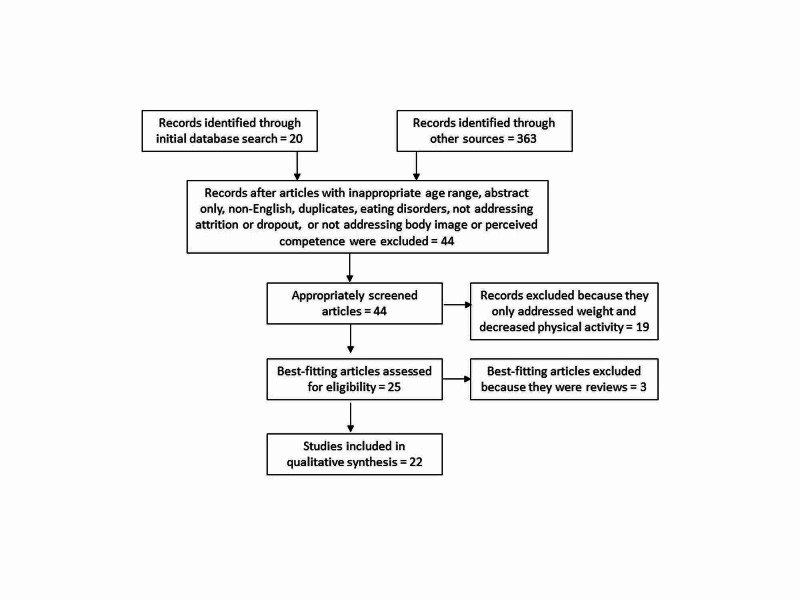
Preferred Reporting Items for Systematic Reviews and Meta-Analyses (PRISMA) Study Selection 2009 Flow Diagram

Three hundred eighty-three articles met the initial criteria. Of those, two were excluded because they were not in English, 11 were excluded because they were only abstracts, seven were excluded because they were related to eating disorders, 37 were outside the age range, 90 did not address body image or perceived competence, and 192 did not address attrition or dropout from sports. From the remaining 44 most accurately fitting articles, 19 were excluded because they were related to increased weight being a deterrent of physical activity and they did not add new data to the time continuum, and, lastly, three were excluded because they were reviews (Figure 1).

The age-related continuum of data, viewed across the years of childhood, portrays a greater picture and provides more specific information regarding the temporal nature of interventions than each article alone. A summary of the results is detailed in Table 1. Mean age was included if the age range of participants was not provided.

**Table 1:** Summary of the Articles Organized by Age to Reflect the Changes in Body Image Children Experience as They Develop. *year of publication

AUTHORS	YEAR*	DEMOGRAPHICS	CAUSE OF ATTRITION
Howie et al. [12]	2019	Birth – 5 yrs	Girls and boys who were breastfed, attended child care, were taller, and without behavioral problems consistently participated in physical activity. Girls with previous injury or parental concern regarding health dropped out, and boys who were premature, non-injured, or with dysfunctional families were dropouts.
Barnett et al. [13]	2016	19 mos – 5 yrs	The amount of time in moderate-to-vigorous physical activity is important to subsequent actual and perceived motor competence.
Crane & Temple [8]	2015	Mean 5 yrs, 7 mos	Kindergarten children’s participation in physical activity is not yet influenced by their perceptions. Object control skills predicted engagement as well as their perceptions of competence, but perceptions did not in turn mediate the relationship between motor proficiency and physical activity.
LeGear et al. [14]	2012	Mean 5 yrs, 9 mos	Children have positive perceptions of their physical competence. Modest relationships between perceptions of competence and motor skill proficiency suggest children are beginning to make self-judgements.
Barnett et al. [15]	2015	4 – 8 yrs	Actual object control was positively associated with perceived object control, not the amount of time in moderate-to-vigorous physical activity. Girls have poorer object control skills.
Slykerman et al. [16]	2016	5 – 8 yrs	Boys have higher actual and perceived object control skills and were more active than girls. Locomotor skills for girls were a predictor of activity.
Estevan et al. [17]	2019	5 – 11 yrs	Highly capable children, with high perceptions, exhibited higher physical activity and were more likely to be of normal weight.
Poulsen et al. [18]	2011	6 – 11 yrs	Overweight children had lower physical abilities and lower self-concept at very young age.
Stodden et al. [3]	2008	9 – 11 yrs	Perceived motor skill competence influences the development of actual motor skills and physical activity. Ability to compare oneself to others significantly affects attrition.
Jensen & Steele [10]	2009	5th or 6th grade	Weight criticism in girls with body dissatisfaction negatively affects participation in physical activity.
Schmalz & Davison [19]	2006	11 – 13 yrs	Overcoming stereotypes, boys and girls playing cross-gendered sports, linked with higher self-esteem.
Hausenblas et al. [20]	2002	Mean 12.61 yrs	Body dissatisfaction linked to low fitness levels, and body image concerns prevail in the absence of weight issues.
Slater & Tiggemann [2]	2011	12 – 16 yrs	Teasing and body image concerns reduced rates of participation.
Field et al. [9]	1999	5th – 12th grade	Girls exercised to lose weight because they perceived they were too big by seeing magazine images.
Ommundsen & Vaglum [21]	1997	12 – 16 yrs	Low coach-evaluated competence and low perceived competence result in high dropout in 12- to 13-year-olds. In 14- to 16-year-olds, low perceived competence alone is a direct precursor to dropout.
Boiche & Sarrazin [22]	2009	Mean 14.6 yrs	Perceived competence, level of satisfaction, personal value, parents’ investment, mastery environment by coaches, and good teammates prevent dropout from sport.
Monsma et al. [23]	2006	12.8 – 22.3 yrs	The premenstrual skaters had lower self-esteem, global physical self-concept, and appearance scores, despite having the preferred body shape.
Barnett et al. [24]	2008	14.2 – 18.3 yrs	Developing a high perceived sports competence through object control is important in determining adolescent physical activity.
Barnett et al. [25]	2011	14.2 – 18.3 yrs	Perceived sports competence is part of a positive mediator between object control proficiency and locomotor skill acquisition and physical activity levels.
Carlman et al. [7]	2013	School-aged children	Attrition due to “not fun” and desire more time for leisure activity, friends, and school. Those with more competency switched sports.
Harter [26]	1978	Children	Perceptions serve as an important mediator, maintaining or increasing a child’s motivation.
Williams & Cash [27]	2001	Mean 21.7 yrs	Weight training improved body image, social anxiety decreased, and physical activity increased.

Youngest athletes (data on ages - birth to 5 years and 7 months)

A positive relationship between motor skill performance and physical activity is not as apparent in the youngest athletes [12]. There was no correlation between gross motor skill acquisition as babies and toddlers and sports participation later in life [12].

Children under the age of 7 years demonstrate an inflated self-perception of their motor skill competence. Younger children are eager to participate in activities whether or not they are competent in those activities [3]. High perceptions of competence contribute to increased physical activity and act as a valuable driving force to the acquisition of motor skills [3]. At a young age, participation in physical activity is not yet influenced by perceptions [28]. At a mean age of 5 years and 7 months, children’s activity participation did not seem to be affected by their perceptions, but, as they crept closer to 6 years (mean age, 5 years and 9 months), perception of their skills began [14,28]. 

Despite performing slightly lower on a gross motor development test, kindergarten-aged children still perceived their abilities as high [14]. When assessing perceptions of skills using locomotor skills, kindergarten girls had higher perceived physical competence than boys; this was a unique finding, as perception of skills is typically lower in girls [14]. Results point to early kindergarten as a window of opportunity to improve children’s fundamental movement skills, especially for girls, as this may lead to greater involvement in activity later in life [14].

In the young population (19 months-5 years), higher levels of exposure to physical activity correlate with increased sports participation [13]. Results of more than three years of longitudinal data found that higher levels of moderate-to-vigorous physical activity (accelerometry) at 3.5 years were associated with actual locomotor skill and perceived total skill at 5 years [13]. As children age, modest relationships between perceptions of competence and motor skill develop [14,15]. There is convincing data that physical activity and actual motor skill competence are associated in children aged 4 years and older [6,13]. 

Later childhood ages (data on ages - 5 years and 9 months to 11 years of age)

As children age (4-8 years), actual object control results in greater physical activity [6]. It has been hypothesized that children with better motor competence participate in higher levels of physical activity and that this in turn helps to further develop higher actual and perceived motor competence [13]. As early as 6 to 10 years of age, children start to make self-judgements of their abilities compared with peers [14]. Around 7 to 10 years of age, children begin to more accurately compare their skills with those of their peers, and their perception of their abilities, whether or not they are being measured, can result in attrition [16,28]. Actual rather than perceived skill was more indicative of physical activity in the Slykerman et al. cohort [16], therefore indicating that the relationship is only just emerging at this age. Between 9 and 11 years, children develop a more accurate perception of their skills in comparison with others. 

Body mass index (BMI) becomes a major determinant in body image and continuation of physical activity between 6 and 11 years of age [18]. Children who were overweight or obese struggled with fundamental movement skills, bilateral motor coordination, body strength, balance, speed, and agility [18]. Overweight children have lower perceptions of themselves regarding physical abilities and are less likely to continue participation in sports and other active leisure-time pursuits [18]. This is an extremely vulnerable time for children. Poulsen et al. [18] reported on a group of young children 6 to 11 years of age (mean age 8.75 years) who already developed poor physical abilities and self-concept. Equally disheartening was the cohort of 5th and 6th graders (mean age 10.8 years) who reported experiencing high levels of body dissatisfaction [10]. Low body image correlated positively with physical activity, but, when the girls with low body image were criticized for their weight, their participation decreased [10].

Adolescent athletes (ages 12 and older)

During the adolescent years, the ability to perform object control skills (catching, throwing, kicking) relates to athletes’ perceptions of their athletic competence [24]. Actual competence is needed for perceived competence, which is the factor affecting participation in physical activity [24]. The previous common understanding of sports attrition is the positive relationship between motor competence and physical activity across childhood [4]. But results have shown, especially in adolescents, perception of skills may be lower than actual skills [11,20]. In a cohort of 12- to 16-year-old male soccer players (mean = 14.5), low perceived competence directly affected dropout from soccer, whether or not the adolescents felt that soccer was important [21].

In adolescence, body image, teasing, and gender identification issues become significant determinants of continuation of physical activity, more so than actual skill [2,20]. Motor competence is both a precursor and a consequence of weight status and demonstrates an inverse relationship across childhood and adolescence with BMI [4]. Fitness levels and BMI often display a negative inverse association [4,11]. Subjects also report body dissatisfaction and poor body image as major determinants in enjoyment and fitness levels [2,9,18,20,28]. Hausenblas et al. [20] discovered body dissatisfaction was a greater deterrent of physical activity than BMI. Children also had poor body image concerns with a healthy BMI, but a higher BMI did relate to lower body image [20].

Adolescents are teased about their weight and coordination [2]. They are also becoming aware of societal pressure to measure up to magazine pictures or elite athletes in their sport [9]. During this time, self-perceptions appear to deviate from skill. Even the most unsuspecting athletes, like the premenstrual female figure skaters who still maintain an ideal body type, become inappropriately sensitive and negative toward themselves [23]. The more frequently girls observed images in fashion magazines, the more dissatisfied they were with their own body [9]. In the case of magazines, girls were more inclined to lose weight, go on a diet, exercise to lose weight, improve their body shape, or exercise because of an article [9]. Gym attendance appears to negatively affect their perception of themselves as does gaining weight [2,18]. Their perceptions are lower than their skills, and this results in them opting out of physical activity because they perceive they are not as competent as their peers [11,20]. If we are able to keep this population in sports until they are slightly older children and adolescents, then physical activity and weight positively correlate again [11]. Evidence reveals that educational fitness programs, where children learn about the effects of physical activity, can improve perceived and actual fitness levels in young people [6].

Gender identification

At a very early age, sports participation differs between boys and girls depending on previous injury. Previously injured boys are more likely to continue participation compared with previously injured girls who are more likely to drop out [12]. This may be connected to boys being viewed as tougher and can “take a hit,” whereas parents are more protective of injured girls [12].

Pubertal development is linked with adolescents’ perceived physical self-concept, and boys and girls experience a different degree of physical self-concept depending on the type of sport in which they participate [19]. Girls more frequently felt that people were staring at them during physical activity. Girls are more likely to report being made fun of or laughed at because of how they looked [2]. They are concerned that a sport may make them less feminine or too feminine [2]. Girls also experienced more teasing for being uncoordinated and being called names because of their weight than boys [2]. Especially during adolescence, girls experience body dissatisfaction and express a desire to be thinner [2]. This body dissatisfaction affects the propensity to participate in sports and other physical activities [2,11].

Interestingly enough, there was a link between social stereotypes of gender and sports participation. Both boys and girls who participated in more cross-gendered sports (boys in ballet and girls in football), in addition to gender-typed sports, have an improved body image and self-concept than those who only participate in gender-typed sports [19]. Particularly in girls, those who participated in skateboarding and rollerblading had an improved sense of self [19].

Discussion

The literature supports the hypothesis that body image and perceived competence act as mediators for physical activity in older children and adolescents. The results of this study suggest participants’ positive perception of their physical competence and image positively correlates with participation in physical activity. Perceiving they are not good enough, or not as good as they hoped, or lacking skill improvement was strongly linked to their discontinuation of physical activity [8]. The model conceived by Harter [26] in 1978 is supported: children who perceive competence and have high self-esteem are more likely to participate. Most remarkable was that the participants’ perceptions of competence predicted the extent to which they valued the activity, despite actual competence not being measured [8]. Strategies to enhance physical self-perceptions in children and adolescents may assist in efforts to promote physical activity [6]. Sports attrition appears to be moderately associated with fitness and at higher physical activity levels was associated with higher levels of general physical self-concept [6]. The astonishing phenomenon identified by this research was the age at onset of negative body image [18].

Children start to perceive competence as early as 6 years of age. Age 5 years may be the window of opportunity to improve acquisition of fundamental movement skills: object control particularly and locomotor skills [13,14]. Evidence has shown that interventions to increase physical activity early in school years are effective in the development of motor skills even at a young age [29]. Five years of age or starting in preschool is the prime time for improving children’s skill competence because children at this age view themselves as more competent than they are capable [14]. They are not able to accurately view their competence, and their skill levels are relatively equal between males and females. Greater exposure to activities in youth has been shown to lead to greater sports participation in children [6,24]. The key to improving youth sports participation may be capitalization on this time frame in children’s lives when they have an inflated self-perception. By exposing children to a wide variety of fundamental movement skills and by spending a longer duration of time on the development of physical literacy, children may perceive their abilities as higher later in life. Also, previous physical activity appears to have more influence on children’s perceptions than current physical activity [13]. Therefore, if the initiation of physical activity begins when children are less self-conscious, their success rate might be higher later in life. Understanding the effects of age on participation in physical activity can have profound effects later in these children’s lives. 

Fundamental movement skill ability in older children and adolescents is positively associated with engagement in physical activity [24]. A positive relationship exists between competence, physical activity, weight, strength, and endurance [4]. In older children, the relationship between object control skill and physical activity becomes more important. Movement skill interventions are effective, and it is likely that their benefits can be sustained. There is opportunity to improve children’s fitness by improving their perceptions of sports competence, especially targeting object control skills [24]. Interventions at a young age appear to have a “flow-on effect” on physical activity later in childhood. Children who are more proficient in object control are more likely to become active adolescents [30]. There is evidence that intervention at a young age can produce significant and meaningful improvements in motor skill acquisition and positively influence perceptions of competence and self-esteem [14].

Perceived competence plays an even greater role in motivation for physical activity, possibly more so than actual competence, as children age into adolescence [21]. Interventions that stressed motor skill activity appear to be the best for improving perceived competence and enjoyment in physical activity [31]. There is opportunity for the reintegration of adolescents into physical activity. Educational programs and strength-based weight training have been effective at promoting a healthier body image and increasing physical activity in adolescents [27]. Exercise programs based on fundamental movement skills and motor coordination skills are the most effective in improving desire to participate in physical activity in the overweight and obese youth population [31]. Coaches play an important role in young players’ participation by fostering an individual mastery climate among their teams [5,21]. Better understanding of the needs of these adolescents may not only provide them better care and programs but may improve the success of those programs available.

Limitations in identifying all references because of synonyms used for the searchable items were challenged by including those synonyms in searches. Bias toward articles that provided novel information may have minimized the appearance of how many articles support the effect of BMI on participation in physical activity. Further research is needed on what forms of physical activity, object control versus locomotor skills, for example, are recommended during the “window of opportunity” to improve development of physical literacy. 

## Conclusions

Literature reveals that body image is significantly associated with physical activity in youth. The very young demonstrate an inflated perception of motor skill competence, which may be a valuable window into the acquisition of fundamental movement skills and the eventual development of physical literacy. Actual skill, particularly object control, does correlate with physical activity as children age, but there may be an opportunity to influence perceived competence by promoting physical activity in the very young. There does appear to be success in programs that place emphasis on education and improving body image, decreasing teasing and comparison with others, where children are able to succeed and personally improve. These programs may assist in the reintegration of adolescents into physical activity.
